# Development of a Gold Nanoparticle‐Assisted Multiplex Polymerase Chain Reaction Assay for Selected Hospital‐Acquired Infections‐Associated Bacterial and Fungal Pathogens

**DOI:** 10.1002/open.70294

**Published:** 2026-09-15

**Authors:** Kubra Aslan, Emre Erden Kopar, Serap Evran

**Affiliations:** ^1^ Chemistry Department Faculty of Science Ataturk University Yakutiye Erzurum Türkiye; ^2^ Biochemistry Department Faculty of Science Ege University Bornova İzmir Türkiye

**Keywords:** hospital‐acquired infections (HAI’s), multiplex PCR, nano‐PCR, pathogen detection

## Abstract

In this study, development and optimization of a multiplex PCR assay for the simultaneous amplification of selected bacterial and fungal targets were performed. Source of hospital‐acquired infections‐specific, (*Staphylococcus aureus*, *Klebsiella pneumoniae*, *Pseudomonas aeruginosa*, *Candida albicans*, *Fusarium oxysporum*, and *Scedosporium prolificans*) primer sets targeting conserved genomic regions were systematically evaluated for potential nonspecific interactions, cross‐reactivity, and structural stability using in silico tools and empirical analyses. Multiplex PCR conditions were optimized using genomic DNA from reference strains under controlled laboratory conditions, with particular emphasis on annealing temperature, MgCl_2_ concentration, and primer balance. In addition, the effect of gold nanoparticles (AuNPs) on amplification performance was investigated. The inclusion of 1.0 nM 10 nm AuNPs enhanced overall amplification efficiency, with a more pronounced improvement observed for fungal targets. Overall, this study establishes an analytical proof of concept for a nanoparticle‐assisted multiplex PCR configuration capable of simultaneously amplifying multiple bacterial and fungal targets in a single reaction.

## Graphical abstract

Gold nanoparticle‐assisted multiplex PCR enables the simultaneous detection of six major hospital‐acquired infection‐associated pathogens by improving amplification performance and assay specificity, providing an analytical proof‐of‐concept for rapid molecular identification.

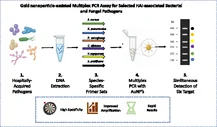

## 1 Introduction

Hospital‐acquired infections (HAIs, or healthcare‐associated infections), also called nosocomial infections, are infections that occur due to medical care in hospitalized patients within 48 h to 30 days after receiving healthcare [1]. Timely intervention is essential for effective treatment and for reducing antimicrobial resistance, prolonged hospitalization, disability, mortality, and outbreaks. To address this need, a multiplex PCR method, in which primers allow the detection of several organisms by a single assay and thereby provide cost‐effectiveness and shorter time to diagnosis, was developed to detect HAI‐causative bacterial (*Staphylococcus aureus*, *Klebsiella pneumoniae*, and *Pseudomonas aeruginosa*) and fungal pathogens (*Candida albicans*, *Fusarium oxysporum*, and *Scedosporium prolificans*). By enabling the simultaneous detection of multiple organisms in a single assay, multiplex PCR provides a cost‐effective approach and shortens the time to diagnosis [1, 2, 3, 4, 5]. The bacterial species *S. aureus*, *K. pneumoniae*, and *P. aeruginosa* are among the most common multidrug‐resistant pathogens associated with HAIs, often requiring urgent identification for appropriate treatment [6, 7, 8, 9]. *S. aureus* includes methicillin‐resistant *S. aureus*, a major concern in HAIs, while *K. pneumoniae* is frequently associated with extended‐spectrum beta‐lactamase and carbapenem‐resistant strains [10]. *P. aeruginosa* exhibits intrinsic resistance mechanisms and can develop resistance to multiple antibiotic classes, making rapid detection crucial for infection control [11]. Similarly, *C. albicans*, *F. oxysporum*, and *S. prolificans* are clinically important opportunistic fungi associated with HAIs. *C. albicans* is the leading cause of hospital‐acquired candidemia, whereas *F. oxysporum* and *S. prolificans* exhibit intrinsic resistance to many antifungal agents, making early detection important for appropriate treatment [12]. The six target pathogens were selected because they are among the most prevalent causes of HAIs, are frequently associated with antimicrobial resistance, and collectively provide a representative panel for the development and evaluation of the proposed multiplex PCR assay.

Multiplex PCR enables the simultaneous amplification of multiple DNA targets in a single reaction, providing rapid and cost‐effective pathogen detection. However, successful multiplex PCR requires careful optimization of primer design and reaction conditions to minimize nonspecific amplification and primer competition [13, 14, 15, 16, 17, 18, 19, 20, 21, 22, 23, 24, 25]. Recently, several nanomaterials, including gold nanoparticles (AuNPs), quantum dots, carbon nanotubes, and magnetic nanoparticles, have been investigated as PCR‐enhancing additives, with nanomolar concentrations reported to improve PCR specificity, sensitivity, and amplification yield [13, 26, 27, 28, 29]. Beyond conventional optimization parameters (e.g., primer concentration, MgCl_2_ concentration, and annealing temperature), nanomaterials have recently attracted considerable attention as PCR enhancers. Among the various nanomaterials investigated for PCR enhancement, AuNPs have received the greatest attention because of their excellent biocompatibility, chemical stability, ease of synthesis, and reproducible effects on PCR performance [30, 31, 32]. Therefore, AuNPs were selected in the present study as a well‐established nanomaterial for optimizing multiplex PCR amplification.

The methodological novelty of the present study lies in the development of a six‐target multiplex PCR configuration that simultaneously combines bacterial and fungal genomic targets and in the systematic evaluation of 10 nm AuNPs within this complex reaction system. In contrast to previously reported nano‐PCR assays that have predominantly focused on single‐target or duplex amplification, the present assay contains twelve primers and six genomic DNA templates with different amplicon sizes and genomic complexities. The study further evaluates the concentration‐dependent effect of 10 nm AuNPs on multiplex PCR performance to establish an optimized six‐target nano‐PCR assay.

## 2 Material and Methods

### 2.1 Materials

Bacterial strains used in this study (*S. aureus* subsp. *aureus* ATCC 6538, *P. aeruginosa* ATCC 27853, *K. pneumoniae* BAA‐2473) were purchased from the American Type Culture Collection (Manassas, Virginia, USA). *Taq* DNA Polymerase (recombinant) (5 U/μL), supplemented with 10X *Taq* Buffer with (NH_4_)_2_SO_4_, 2.5 mM MgCl_2_, and ultrapure sterile DNase/RNase‐free water, was purchased from Thermo Fisher Scientific (Waltham, Massachusetts, USA). The PCR primers (Table 1) on a 50 nmol scale were synthesized by Sentegen Biotechnology (Ankara, Türkiye). 10 nm AuNPs in 0.1 mM PBS (Cat. no:752584) were purchased from Sigma Aldrich (St. Louis, Missouri, USA). All AuNP dilutions were prepared with 0.1 M PBS (80.0 g NaCl, 2.0 g ‍KCl, 21.7 g Na_2_HPO_4_ · 7H_2_O, 2.59 g KH_2_PO_4_), a surfactant‐free buffer, sterilized, and diluted to a 0.1 mM concentration. 50 ‍bp DNA Ladder was purchased from NEB (Frankfurt, Germany), and a 1 kbp GeneRuler Express DNA ladder was purchased from Thermo Fisher Scientific. The filtered and autoclaved stock 5X TBE (54.0 g Tris base, 27.5 g boric acid, 20 mL 0.5 M EDTA [pH‍ 8.2–8.4] for 1000 mL) buffer was prepared for both agarose gel preparation and electrophoresis running buffer. Primer and DNA stock solutions and dilutions were prepared with sterile, DNase/RNase‐free TE buffer (15.8 g Tris·Cl, 2.9 g of EDTA [pH 8] for 1000 mL). All other chemicals were of analytical grade.

**TABLE 1:** The sequences of the primers.

Organism	Gene region	Primer sets	Amplicon size, bp	References
*C. albicans*	*CaYST1*	CaF‐5′‐AAGTATTTGGGAGAAGGGAAAGGG‐3′	310	[33, 34]
		CaR‐5′‐AAAATGGGCATTAAGGAAAAGAGC‐3′		
*F.* *oxyspor* *um*	*ITS4* and *ITS5*	FoF‐5′‐ACATACCACTTGTTGCCTCG‐3′	340	[25]
		FoR‐5′‐CGCCAATCAATTTGAGGAACG‐3′		
*S. prolificans*	ITS2‐18S rRNA	SproF′‐5′‐GCTACTACCGATTGAATGGCTC‐3′	642	In this study
		SpoR′‐5′‐GAATAGAGGGTTTGACGGCTG‐3′		
*S. aureus*	*ileS*	SaF‐5′‐CATACAGCACCAGGTCACGGGGAA‐3′	227	[35]
		SaR‐5′‐GTTCTCCAGTCGTGTGGATAGC‐3′		
*P. aureoginosa*	*oprL*	PaF‐5′‐ATGGAAATGCTGAAATTCGGCAAATTTGCTGC‐3′	504	[36]
		PaR‐5′‐CTTCTTCAGCTCGACGCGACGGTTCTGAG‐3′		
*K. pneumoniae*	*ITS*	KpF‐5′‐ATTTGAAGAGGTTGCAAACGAT‐3′	130	[37]
		KpR‐5′‐TTCACTCTGAAGTTTTCTTGTGTT ‐3′		

A glycerol stock (−80 °C, 20% (v/v)) of bacterial cell suspensions prepared from a starter culture of lyophilized form were inoculated into LB medium (tryptone 10 g/L, yeast extract 5 g/L, 10 g/L NaCl‐sterilized, 121 °C, 1.5 atm for 20 min), incubated at 165 rpm for 18–24 h on an orbital shaker as first activation and this step was repeated twice. The genomic DNA was isolated by using a GeneJET Genomic DNA purification kit from Thermo Fisher Scientific.The purity of the DNA template was checked through 1% (w/v) agarose gel electrophoresis. The concentration of the isolated DNA was calculated by absorbance measurement at 260 nm using a BioTek Epoch 2 microplate spectrophotometer (Agilent Technologies, Santa Clara, California, USA). Fungal genomic DNAs were a kind gift from Dr. María José Buitrago Serna from Instituto de Salud Carlos III, Madrid/Spain (*C. albicans* CNM‐CL 8701, *F. oxysporum* CNM‐CM 3197, and *S. ‍prolificans* CNM‐CM 1627).

### 2.2 Analysis of Primer Annealing Temperature and Primer Specificity

Table 1 shows the primer sequences and amplicon’ sizes. The National Center for Biotechnology Information (NCBI) database was used to examine the possibility of nonspecific amplification of other microorganism gene regions beyond targeted genes. The OligoAnalyzer Tool of Integrated DNA Technologies (IDT) was used to examine the tendency of primer pairs to form secondary structures. Stocks of 100 µM of synthesized primers were dissolved in TE buffer, and 10 µM working solutions were prepared from each primer set.

The melting temperature (*T*_m_) of primers was calculated, and gradient PCR was performed to optimize the annealing temperature. Conventional PCR reactions were set up in 50 µL using 25 pmol of forward and reverse primers, 50–100 ng of template DNA, 200 µM of each dNTP, and 0.75 U of *Taq* polymerase enzyme. The PCR program consisted of an initial denaturation at 95 °C for 3 min, followed by 30 cycles of denaturation at 95 °C for 30 s, 30 s of annealing at varying temperatures, elongation at 72 °C for 1 min, and final elongation at 72 °C for 5 min.

### 2.3 Agarose Gel Electrophoresis

Conventional singleplex PCR products were analyzed using 1% ‍(w/v) agarose gel electrophoresis, and multiplex PCR products were analyzed using 2% (w/v) agarose gel electrophoresis. PCR samples were loaded onto the gel, and electrophoresis was run at 120 V until the dye line was ≈75%–80% down the gel (~45 min). After electrophoresis, the gel was placed onto a UV transilluminator and visualized at 365 nm UV light. The efficiency of each PCR reaction was analyzed by comparisonwith a DNA marker, and the band intensities were measured using ImageJ software and were used only for relative comparison of amplification efficiency among the tested conditions. No normalization procedure was applied.

### 2.4 AuNP‐Assisted Multiplex PCR and Optimization Studies

A 50 µL reaction was designed for multiplex polymerase chain reaction, which included primers at equimolar concentrations of 10 pmol from each, 10 ng of each template DNA, 10 µM from each dNTP, and 2.5 U of *Taq* polymerase enzyme. The PCR program consisted of initial denaturation at 95 °C for 3 min, followed by 30 ‍cycles of denaturation at 95 °C for 30 s, primer annealing between 50 and 55 °C (50.0–52.1–53.7–55 °C) for 30 s, elongation at 72 °C for 1 min, and final elongation at 72 °C for 5 min. Gradient PCR was set up with varying concentrations of MgCl_2_ in the range of 1.5–4 mM, incremented by 0.5 mM. Upon the achievement of amplification of the six targeted genes, a multiplex PCR with AuNPs and one control reaction without AuNPs was performed. AuNPs were diluted to a final concentration in the range of 0.5–3.0 nM, incremented by 0.5 nM, and this step was repeated between 0.5–1.0 nM, incremented by 0.15 nM for accurate concentration [38, 39].

## 3 Results and Discussion

### 3.1 Study Design and Preanalyses

The *ileS* gene (227 bp) of *S. aureus* [35]; *oprL* gene (504 bp), encoding a peptidoglycan‐associated protein of the outer cell membrane of *P. aeruginosa* [40]; *ITS* region (130 bp) of *K.‍‍‍ pneumo‍niae* ‍[37]. ITS2‐18S rRNA (642 bp) of *S. prolificans*; *CaYST‐1* gene (310 bp) encoding a ribosome‐associated protein of *C. ‍albicans* [33]; and ITS4–ITS5 region (340 bp) of *F. oxysporum* are the targeted gene regions in this study for multiplex PCR assay development. Important factors to consider when deciding such vital parameters are oligonucleotide specificity to nucleic acid sequence and the detectability of amplicons after PCR. The specificity of the primer sequence can be preanalyzed by using gene databases for the complementarity to any genes other than the desired one. The selection criteria of the conserved genes were the number of reported successful amplifications at the species level as well as the sequential amplicon sizes that enable them to be differentiated on an agarose gel, ex vivo [40, 41]. Oligonucleotide sets targeting these regions were analyzed on the NCBI database by simply copying the primer sequence to determine primer specificity and homologies. BLASTn results showed that all but one primer set exhibited 100% similarity to the desired gene of the respective microorganism and no match with other microorganisms, neither studied in this paper, was found, but targeted. However, the primers used to detect *K. pneumoniae* target a conserved ITS region, which in silico analysis shows may also be present in species such as *K. variicola* and *K. quasipneumoniae*. Primer sets targeting the conserved gene regions were selected from the literature; only primer pairs to detect *S. ‍prolificans* were designed in this study. The balance between the *T*_m_ of primer pairs, the G–C% content of the sequences, and primer lengths was also checked.

Genes, which can be protein‐coding or nonprotein coding sequences, may be responsible for providing virulence factors or antimicrobial resistance to the harboring organisms and provide a useful tool for researchers to use as an identification marker of the microbial species [42, 43, 44]. Amplification of the 16S rRNA genes is a useful method for identifying bacteria at the species level, as this region is usually highly variable among species [44, 45, 46]. Similarly, the use of internal transcribed spacer regions of rRNA has been suggested by the International Fungal Barcoding Consortium in fungal identification [47, 48, 49]. A wide variety of conserved genes have been targeted via synthetic oligonucleotides, depending on limitations and desired properties of a method, as long as the sequence preserves its function of interest. For instance, depending on subspecies, it is possible to identify *S. ‍aureus* via the mecA gene responsible for facilitating resistance to β‐lactam antibiotics, the orfX gene related to methyl transferase activity during RNA processing, or the ileS gene, encoding the isoleucyl‐tRNA synthetase enzyme, that plays an important role in virulence [28, ‍^50^, ^51^]. Similarly, *S. prolificans* identification has been reported by using either a 303‐bp or 176‐bp length gene from the ITS2 region of rRNA [52].

Optimization of multiplex PCR requires empirical adjustment of reaction parameters that influence primer annealing and polymerase performance. Consistent with previous reports, annealing temperature was identified as one of the key determinants of amplification efficiency in the present assay. Therefore, gradient PCR was used to determine the optimal annealing temperature before further optimization of the multiplex reaction.

Multiplex PCR optimization requires empirical adjustment of parameters affecting primer annealing and amplification efficiency. Therefore, gradient PCR was performed to determine the optimal annealing temperature for each primer pair based on their calculated melting temperatures (*T*_m_). Amplification efficiency was evaluated by agarose gel electrophoresis, and band intensities were comparatively scored using ImageJ (Table S1). Based on these results, the annealing temperature range of 50–55 °C was selected for multiplex PCR optimization (see Section 3.2).

### 3.2 Development of Multiplex PCR

**FIGURE 1:**
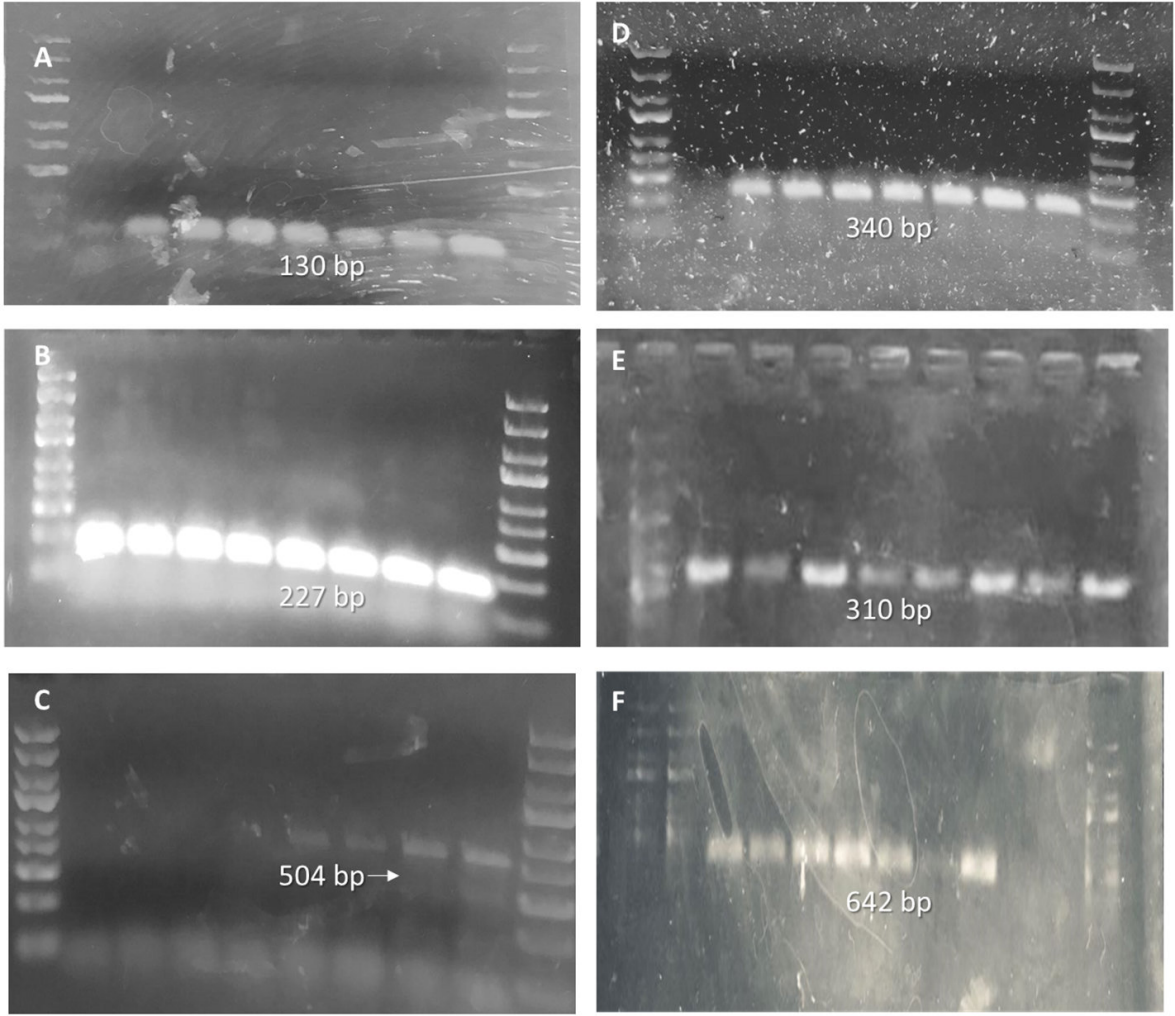
Primer specificity testing by gradient PCR (representative annealing temperatures for each lane are provided in Table S2). (A) ITS region of *K. pneumoniae* (45.0–54.6 °C); (B) *ileS* region of *S. aureus* (54.3–63.0 °C); (C) *oprL* region of *P. aeruginosa* (50.3–59.0 °C); (D) ITS4/ITS5 region of *F. oxysporum* (48.0–57.0 °C); (E) *CaYST‐1* region of *C. albicans* (50.3–59.0 °C); (F) ITS2 rRNA region of *S. prolificans* (50.3–59.0 °C). **M:** GeneRuler Express DNA Ladder (1 kb). Lane numbers correspond to the representative annealing temperatures listed in Table S2.

**FIGURE 2:**
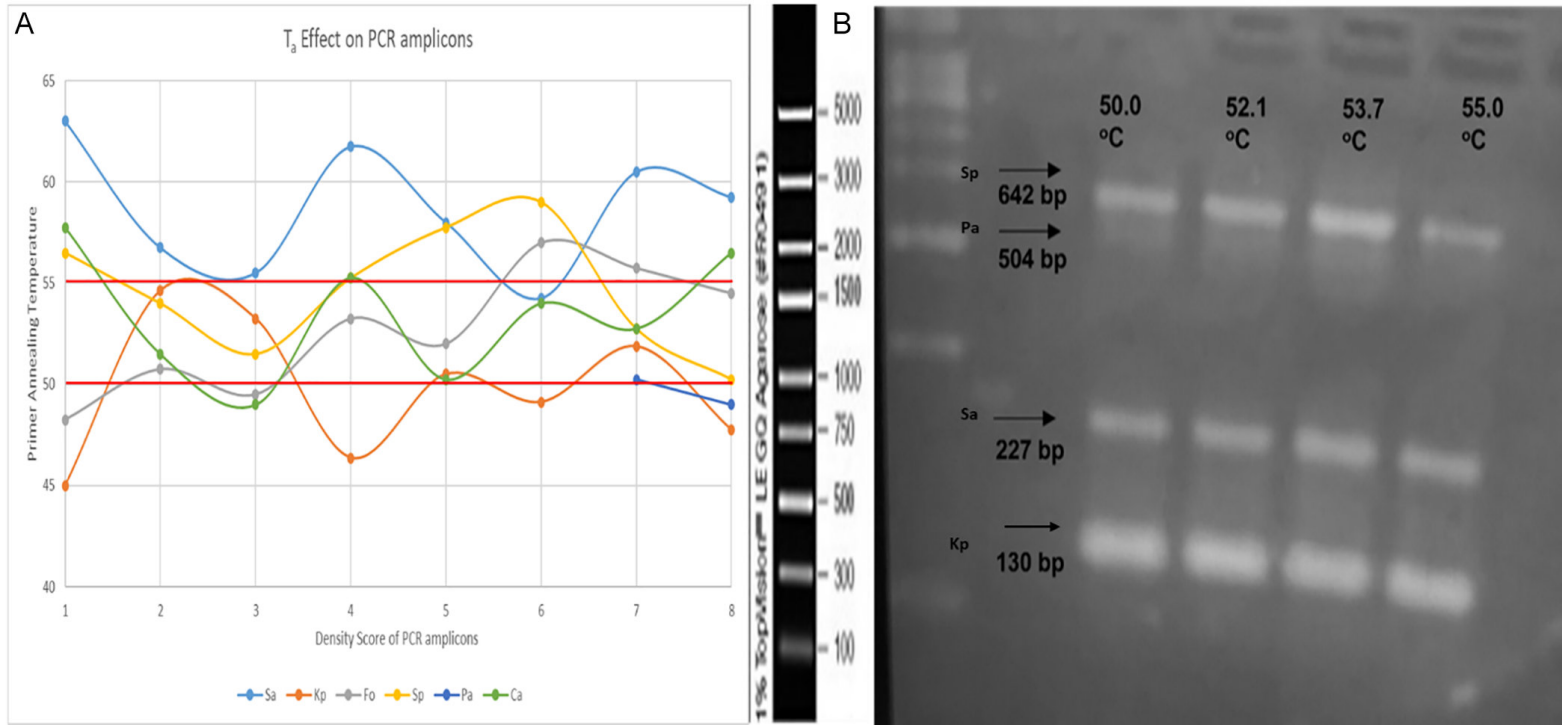
Annealing temperature determination for multiplex PCR; abbreviations: Sa, Kp, Fo, Sp, Pa, and Ca stand for *ileS* from *S. aureus*, *ITS* region from *K. pneumoniae*, *ITS4* and *ITS5* region from *F. oxysporum*, *rRNA ITS2* from *S. prolificans*, *oprL* from *P. aeruginosa*, and *CaYST‐1* from *C. albicans*, respectively. (A) Singleplex PCR density scores of amplicons to 1 and 8, with 1 being the least bright and 8 being the brightest, (B) multiplex PCR amplification results performed at the annealing temperature range with the highest score, Marker name: GeneRuler Express DNA Ladder 1 kb.

In this step, conventional gradient PCR was set up to determine the optimal annealing temperature of each primer pair. The annealing temperature range was derived from the *T*_m_ of primers and 5 °C upper and lower values around the average primer Tms were accepted as limits; eight temperatures within this range were tested for amplification efficiency. Other PCR parameters were adjusted initially as the Thermo *Taq* Polymerase enzyme manufacturer recommended. After amplification, products were analyzed by agarose gel electrophoresis, and according to the results, it was determined that the selected primers were successful in amplifying the genetically conserved region of bacterial and fungal strains (Figure 1). The brightness of amplicons on gel images varied due to different annealing temperatures of the primer; the bands were scored between 1 and 8, with 1 being the least bright and 8 being the brightest. This decision was made based on a visual comparison of bands with markers supported by ImageJ software (open‐source image processing software developed primarily by Wayne Rasband at the National Institutes of Health (NIH) in the United States) analysis (see Table S2). This software measures the density signal of each band detected from the gel image, calculates the area of that signal, and compares the bands in the same gel. The most suitable and narrower annealing temperature range for multiplex PCR was determined from the scores, which point to the range between 50 and 55 °C (Figure 2A). Then, four different multiplex reactions were set up and placed into the thermal cycler by programming the annealing temperature at 50.0, 52.1, 53.7, and 55.0 °C. The concentration of reaction components, except primer concentrations (decreased to 10 pmol), was kept the same. As a result of gel electrophoresis, 53.7 °C was determined to be the annealing temperature of multiplex PCR since higher yields were observed at this temperature; however, the amplification of the ITS4–ITS5 region of *F. oxysporum* and the *CaYST‐1* gene of *C. ‍albicans* could not be determined (Figure 2B). This failure was considered not to originate from the annealing temperature but from other parameters, possibly leading to primer competition.

Many researchers have presented a series of examples of testing various parameters to optimize multiplex PCR [40]. All strategies to optimize multiplex PCR have been performed primarily to regulate polymerase activity and ensure sufficient primer annealing. In general, the specificity of PCR is related to primer annealing to the template, and adjustment of the relevant parameters like annealing time, temperature, and, of course, primer availability to the template is vital in defining the initial PCR condition. Annealing occurs when melted primer successfully complements the template DNA, which is driven by temperature. This binding is dependent on the cooling rate from the denaturation temperature; therefore, the annealing temperature needs to be determined empirically, guided by a theoretically calculated *T*_m_ value derived from DNA’s base content. Gradient PCR is a practical technique that allows one to determine annealing temperature using the least number of steps and minimizing experimental errors.

Because eukaryotic genomic DNA is larger than prokaryotic genomic DNA, the number of double‐stranded DNA in samples and primer access to those templates in reactions will differ, so the reactions were divided into two different settings, bacterial and fungal multiplex PCR, to allow controlled optimizations. According to the results, the *ileS* of *S. aureus*, the *oprL* of *P. aeruginosa*, and the *ITS* region of *K. pneumoniae* were successfully amplified by bacterial triplex PCR, with one nonspecific amplification around 1300 bp and primer dimers. The ITS2‐18S rRNA of *S. prolificans* and the ITS4–ITS5 region of *F. oxysporum* were successfully amplified in fungal triplex PCR. In contrast, the gel band for the *CaYST‐1* gene of *C. albicans* was not amplified, and primer dimers were observed. This result in fungal triplex PCR supported the primer competition between fungal primer pairs. Therefore, primer concentrations of CaF and CaR were increased to 25 pmol, while primers for *S. prolificans* and *F.‍ ‍‍oxysporum* were kept low at 10 pmol [40]. The *CaYST‐1* gene was successfully amplified with the primer concentration increase (data not shown).

**FIGURE 3:**
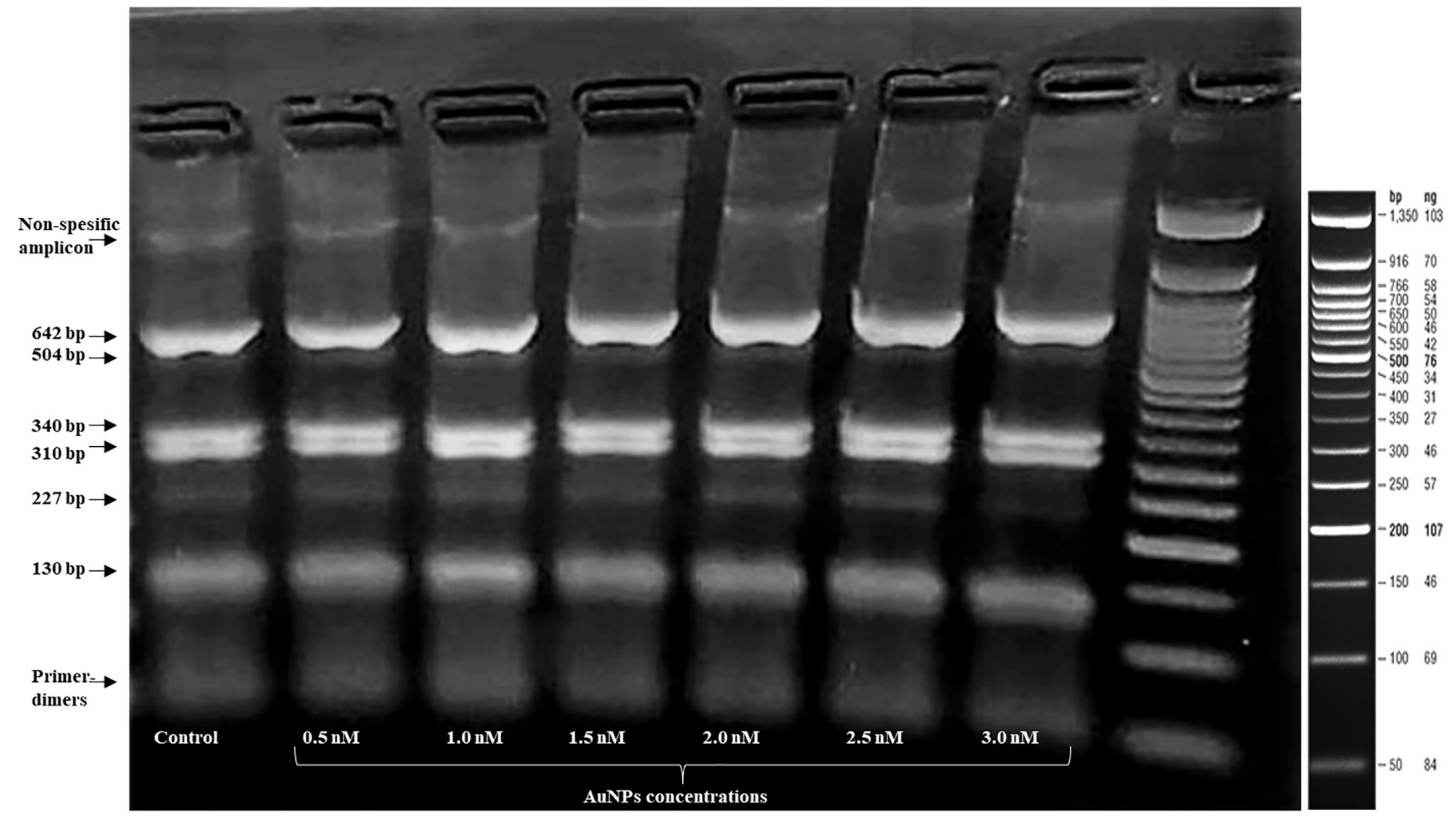
Effect of AuNPs with 0.5 nM increments from 0.5 to 3.0 nM on multiplex PCR, *ileS* (227 bp) from *S. aureus*, *ITS region* from *K. pneumoniae* (130 bp), *ITS4* and *ITS5* region from *F. oxysporum* (340 bp), *rRNA ITS2* from *S. prolificans* (642 bp), *oprL* from *P. aeruginosa*, and *CaYST‐1* from *C. albicans* (310 bp), Marker cat. no.: NEB N3236S.

Bacterial and fungal triplex PCRs were combined by using 10 pmol of each primer except CaF‐CaR and 10 ng of template DNA. The results were analyzed on an agarose gel, showing that all gene regions except the *CaYST‐1* gene of *C. albicans* and the ITS4–ITS5 region of *F. oxysporum* were successfully amplified (Figure 2B). Since the primer annealing was, proceeding to the next step was decided as regulating the polymerase activity. Magnesium chloride is a well‐known divalent cation that interacts with the PCR components like template DNA and dNTPs, increases the melting temperature by stabilizing DNA, and is also required for catalytic activity of DNA polymerase [40, 41, 53]. Inadequate magnesium chloride concentration can cause the PCR failure or result in incomplete amplification. To overcome the failure of PCR for *C. albicans* and *F. oxysporum*, magnesium chloride gradient PCR (1.5–3.0 mM) was set up using the same cycling conditions. As a result, the multiplex amplification of six conserved regions was successful at 3.0 mM magnesium chloride concentration (data not shown). Final product concentrations were determined by visual comparison to the NEB 50‐bp DNA ladder, and the brightness of all amplicons was similar to each other, except for the *oprL* from *P. aeruginosa* and the *ileS* from *S. aureus* genes, which were relatively inefficient. Since among the genomes, that of *P. aeruginosa* has the highest G–C% content (67.2%), therefore, denaturation and annealing times were elevated. However, no significant change in the amplification of the locus was recorded. Primer pairs PaF and PaR were extended 9 and 8 bases, respectively. Still, amplification of this *oprL* remained inefficient, and primer dimer at the bottom of the gel was observed and could not be overcome with PCR parameter optimization studies (see Figure 3 for the formation of these primer dimers even with AuNP optimization conditions). While primer specificity was initially evaluated through BLAST searches and literature‐based design, we acknowledge that the primer pair used for *K. pneumoniae* targets a conserved ITS region that may also be present in closely related species such as *K. variicola* and *K. quasipneumoniae*. This could lead to potential cross‐amplification.

### 3.3 AuNP‐Assisted Multiplex PCR

**FIGURE 4:**
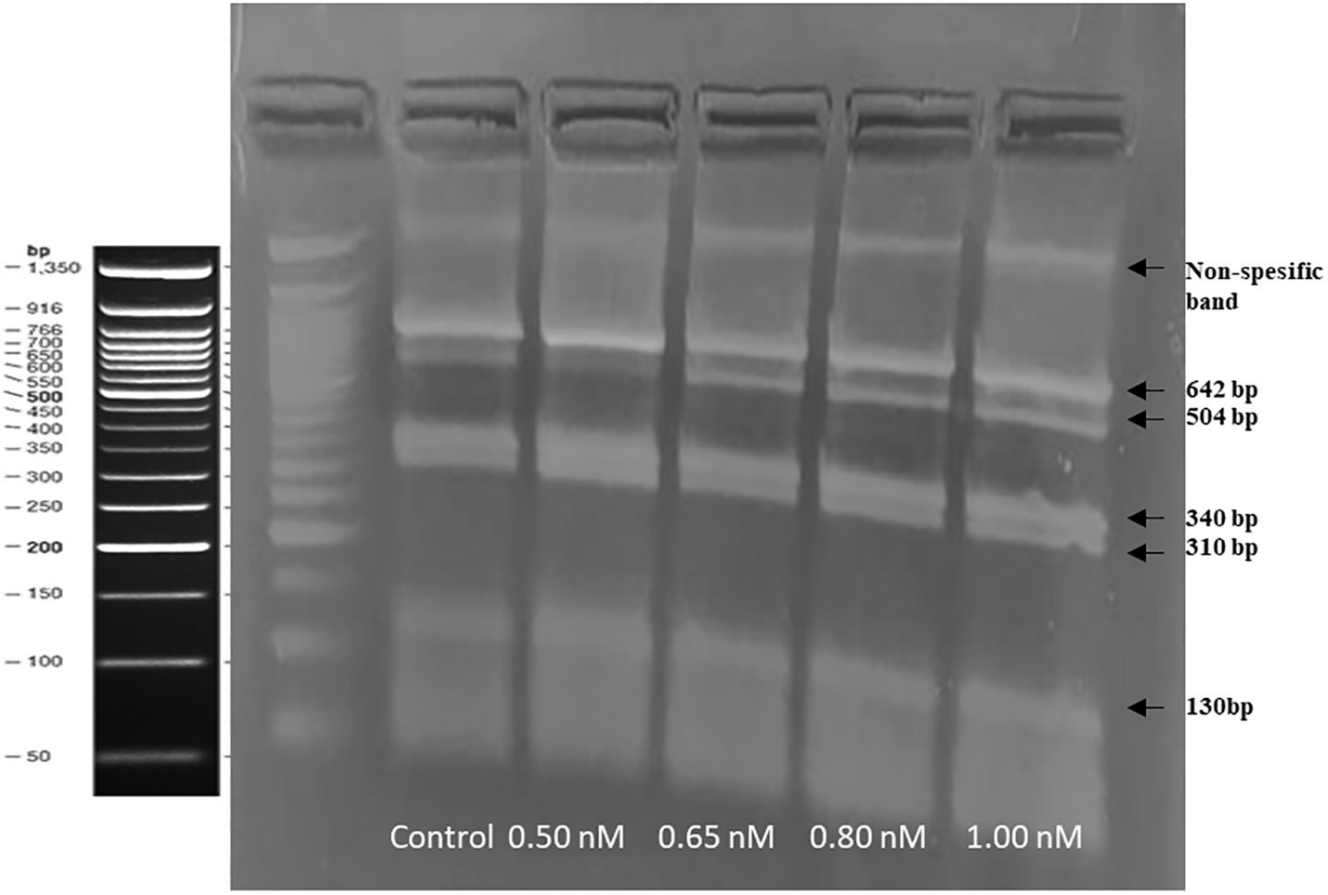
Effect of AuNPs with 0.15 nM increment from 0.5 to 1.0 nM on multiplex PCR, Marker cat. no.: NEB N3236S.

10 nm size AuNPs in the concentration range between 0.5 and 3.0 nM were added to multiplex PCR to investigate the effect of AuNPs on PCR efficiency. The results were analyzed by comparing sample amplicons against a control reaction that was set up without AuNPs. The results showed that all genes were amplified more efficiently in the presence of 1.0 nM AuNPs than in other reactions, including the control group (Figure 3). This result suggested that more amplicons were obtained with 1.0 nM AuNP, in contrast to Li et al. [54], who claimed an inhibitory effect at this concentration. This could be because of the difference in surface interaction of AuNPs between the two studies, as AuNPs in this study have been dispersed in a neutral PBS buffer, whereas Li et al. used citrate stabilization. Negatively charged AuNPs induce the dipole repulsion of nucleic acids, therefore, an inhibitory effect might be observed on PCR. Amplification of the *ileS* region of *S. aureus* was inhibited with the use of 3.0 nM of AuNP in addition to the overall decrease in the amplification yield of other products. The nano‐multiplex PCR was repeated to increase nanoparticle concentration accuracy with 0.5–1.0 nM of AuNPs, to determine if there is a concentration that is more effective in this range. According to the results obtained from this study, 1.0 nM of AuNPs provided more equal amplification of products. Additionally, as the amplicon size ranges from 130 to 642 bp, this platform is suitable for evaluating the claims of Vu et al. [55]. This result contrasted with the finding that AuNPs have a selective favoring for the amplification of small products over larger ones, regardless of specificity [55]. Selective favoring of smaller products over larger products was not observed; however, an increase in fungal amplification over bacterial amplification was observed when 0.5 nM of AuNPs were used (Figure 4). This can be associated with the positive effect of AuNPs on complex templates. A slight decrease in primer dimers was also observed. The improved amplification observed at 1.0 nM AuNPs suggests that careful optimization of nanoparticle concentration may contribute to a better balance among competing primer pairs in complex multiplex PCR reactions. Likewise, the recovery of fungal amplification following primer and MgCl_2_ optimization indicates that reaction performance was primarily influenced by primer competition rather than primer specificity. Although several mechanisms have been proposed to explain the beneficial effects of AuNPs, including improved heat transfer and interactions with PCR components, the present study was not designed to investigate these mechanisms experimentally. Therefore, these explanations should be considered literature‐based hypotheses rather than direct conclusions of this study.

AuNPs are versatile materials because of their chemical and optoelectronic properties, as well as being nontoxic and biocompatible. Their physical sizes, catalytic activities, surface volume ratios, and light absorption properties can be adjusted through the synthesis and modification of favorable surfaces [38]. In addition to the favorable influence of these unique properties, the reversible interactions of AuNPs with biological molecules enable one to regulate and manipulate these interactions. Li et al. [54, 56] were the first study group to demonstrate the effects of AuNPson PCR by performing error‐prone two‐round PCR with 10 nm AuNPs concentrations ranging from 0.2 to 0.8 nM. They demonstrated that 0.4 nM AuNPs reduced nonspecific products and provided bright amplification at a low annealing temperature down to 25 °C in the presence of 0.6 nM AuNPs, while no products were obtained in the absence of nanoparticles [54, 56]. These findings have encouraged PCR researchers to investigate further the impacts of AuNPs on PCR. Another study group claimed that the amplification yield of slower PCR systems (conventional) and quicker systems (real‐time PCR) could be increased by 0.7 nM of 13 nm AuNPs by 5–10‐fold and 10^4^–10^5^, respectively [38]. Huang et al. [57] discovered evidence of AuNPs’ effect on PCR sensitivity. They conducted a real‐time PCR experiment in the presence and absence of 1.6 and 3.2 nM AuNPs (13.2 nm size). Amplification of DNA fragments for 30 cycles was reduced to 25 ‍cycles with both 1.6 and 3.2 nM AuNPs, while no amplification was observed without AuNPs at 25 cycles [57]. As a result, an AuNP‐assisted multiplex PCR condition was defined to detect *C. ‍albicans*, *S. aureus*, *K. pneumoniae*, *P. aeruginosa*, *F. oxysporum*, and *S. prolificans*. This platform is evaluated as a fast, sensitive, and specific method that can be used for the detection of any type of HAI, such as CAUTI, CLABSI, VAP, or SSI, and can be a versatile tool for timely diagnosis and strategy, and also for efficient treatment. Relevant to the findings of this study, it is reasonable to claim that defining one specific AuNP size and a concentration applicable to any PCR is troublesome, meaning an optimization study may need to be conducted before introducing it to the method, since it also has an inhibitory effect under certain conditions. Nevertheless, AuNPs hold the potential to be more versatile compared to their alternatives, such as bovine serum albumin and formamide. Several studies were conducted to investigate the effects of nanoparticles in diagnostic PCR for the detection of airborne biological (bacteria and viruses) particles [58], porcine parvovirus [59], porcine bocavirus [60], encephalomyocarditis virus [61], mink enteritis virus [62], Japanese encephalitis virus (Huang et al. [57]), and porcine epidemic diarrhea virus [63]. To the best of our knowledge, this is the first study that shows the effect of AuNPs in the PCR detection of fungal pathogens. Some studies have examined the mode of action of nanoparticles on PCR, reporting that thermal conductivity and surface interactions provided by nanoparticles arrange the PCR components on their surfaces, accelerate the heating/cooling steps, stabilize ssDNA and dsDNA and regulate the polymerase activity [13, 28, 29, 64].

The present study focused on the optimization of multiplex PCR conditions and the evaluation of AuNP effects rather than comparison with culture‐based methods. Therefore, further studies using clinical samples are required to determine diagnostic sensitivity, specificity, and limit of detection relative to routine methods.

## 4 Conclusions

This study describes the stepwise development and optimization of a gold nano‐multiplex PCR assay for the simultaneous detection of six bacterial and fungal pathogens associated with HAI’s. By targeting conserved genomic regions, the optimized assay successfully amplified all selected targets using genomic DNA from reference strains. The systematic optimization of reaction conditions identified 1.0 nM of 10 nm AuNPs as the optimal concentration, resulting in improved amplification efficiency, particularly for fungal targets. These findings demonstrate the potential of the optimized AuNP‐assisted multiplex PCR configuration as an analytical proof‐of‐concept for simultaneous detection of selected HAI‐associated pathogens.

Certain limitations should be acknowledged. The primer pair used for *K. pneumoniae* targets a conserved ITS region that may also amplify closely related species, including *K. variicola* and *K. quasipneumoniae*, thereby limiting species‐level discrimination. In addition, the assay was evaluated only with reference strains and has not yet been validated using clinical specimens. Therefore, further studies should assess its diagnostic performance using patient‐derived samples and compare it with established routine diagnostic methods.

## Funding

This work was supported by the Ege University Scientific Research Projects Coordination Unit (project number: 17‐FEN‐009).

## Conflicts of Interest

The authors declare no conflicts of interest.

## Supporting information

Supplementary Material

## Data Availability

The data that support the findings of this study are available from the corresponding author upon reasonable request.
