# Supplementary material for: Development of a Gold Nanoparticle‐Assisted Multiplex Polymerase Chain Reaction Assay for Selected Hospital‐Acquired Infections‐Associated Bacterial and Fungal Pathogens
Source: ChemistryOpen. 2026 Sep 15;15(9):e70294. doi: 10.1002/open.70294 (PMC13578920; doi:10.1002/open.70294)
Supplement: Supplementary file 1 [file OPEN-15-e70294-s001.pdf]

SUPPLEMENTARY DOCUMENT TO

# Nano-PCR for the Multiplex Detection of Bacterial and Fungal Pathogens

| Lower Δ G Kcal/mole | SaF | KpF | PaF’ | CaF | FoF | SpF | SaR | KpR | PaR’ | CaR | FoR | SpR | Hairpin | Lower Δ G Kcal/mole | Higher $T_m$(°C) |
|---|---|---|---|---|---|---|---|---|---|---|---|---|---|---|---|
| **SaF** | -4,41 | -5,09 | -8,65 | -5,02 | -5,09 | -6,68 | -6,69 | -,355 | -4,74 | -5,02 | -3,61 | -6,69 | **SaF** | -0,40 | 31,3 |
| **KpF** | -5,09 | -10,94 | -8,98 | -5,84 | -7,04 | -4,41 | -6,53 | -5,47 | -8,65 | -5,36 | -5,84 | -7,18 | **KpF** | -0,07 | 30,5 |
| **PaF’** | -8,65 | -8,98 | -9,44 | -7,32 | -10,1 | -8,26 | -6,6 | -5,47 | -10,2 | -8,51 | -9,26 | -5,84 | **PaF’** | -2,35 | 48,4 |
| **CaF** | -5,02 | -5,84 | -7,32 | -3,89 | -5,02 | -3,9 | -9,76 | -5,46 | -6,72 | -5,36 | -6,97 | -4,38 | **CaF** | 1.25 | 2,1 |
| **FoF** | -5,09 | -7,04 | -10,1 | -5,02 | -3,61 | -6,24 | -6,37 | -4,88 | -5,19 | -8,16 | -6,24 | -6,24 | **FoF** | 0,37 | 16,5 |
| **SpF** | -6,68 | -4,41 | -8,26 | -3,90 | -6,21 | -3,61 | -5,7 | -5,47 | -8,02 | -6,31 | -8,16 | 6,68 | **SpF** | 0,24 | 20,9 |
| **SaR** | -6,91 | -6,53 | -6,6 | -9,76 | -6,37 | -5,7 | -6,6 | -3,55 | -6,53 | -5,02 | -6,31 | -4,95 | **SaR** | -0,40 | 39,2 |
| **KpR** | -3,55 | -5,47 | -5,47 | -5,46 | -4,88 | -5,47 | -3,55 | -5,47 | -8,67 | -7,41 | -7,41 | -4,77 | **KpR** | -1.10 | 40,1 |
| **PaR’** | -4,74 | -8,65 | -10,2 | -6,72 | -5,19 | -8,02 | -6,53 | -8,67 | -10,4 | -6,31 | -6,31 | -6,99 | **PaR’** | -1,04 | 31,9 |
| **CaR** | -5,02 | -5,36 | -8,51 | -5,36 | -8,16 | -6,31 | -5,02 | -7,41 | -6,31 | -5,37 | -6,21 | -4,74 | **CaR** | -0,07 | 26,1 |
| **FoR** | -3,61 | -5,84 | -9,26 | -6,97 | -6,24 | -8,16 | -5,02 | -3,9 | -6,75 | -6,21 | -5,47 | -6,21 | **FoR** | -0,95 | 36,6 |
| **SpR** | -6,69 | -7,18 | -5,84 | -4,38 | -6,24 | -6,68 | -4,95 | -4,77 | -6,69 | -4,74 | -6,21 | -3,61 | **SpR** | 1,04 | 2,0 |

**Supplementary Table S1.** Primer secondary structure formation tendencies

| *Lane* | *16 S rRNA* from *K. pneumoniae* | | *ITS4 &ITS5* region from *F. oxysporum* | | *rRNA ITS2* from *S. prolificans* | | *CaYST-1* from *C. albicans* | | *IleS* from *S. aureus* | |
|---|---|---|---|---|---|---|---|---|---|---|
| A/B/C/D | $T_a$ | Peak Area (px) | $T_a$ | Peak Area (px) | $T_a$ | Peak Area (px) | $T_a$ | Peak Area (px) | $T_a$ | Peak Area (px) |
| 1 | 45.0 | 259 | 48.3 | 0 | 50.3 | 26502 | 50.3 | 429 | 54.3 | 1151 |
| 2 | 46.4 | 3231 | 49.5 | 1817 | 51.5 | 613 | 51.5 | 1913 | 55.5 | 898 |
| 3 | 47.8 | 4627 | 50.8 | 1100 | 52.8 | 10281 | 52.8 | 369 | 56.8 | 633 |
| 4 | 49.1 | 4473 | 52.0 | 2613 | 54.0 | 463 | 54.0 | 3067 | 58.0 | 1109 |
| 5 | 50.5 | 3879 | 53.3 | 2306 | 55.3 | 2659 | 55.3 | 2109 | 59.3 | 1641 |
| 6 | 51.9 | 4537 | 54.5 | 5095 | 56.5 | 407 | 56.5 | 1129 | 60.5 | 1399 |
| 8 | 54.6 | 2544 | 57.0 | 4439 | 59.0 | 2860 | 59.0 | 15732 | 63.0 | 899 |

**Supplementary Table S2.** Image J analysis results of Gradient PCR that is used to determine the optimal range of annealing temperature of Multiplex PCR; *oprL* from *P. aeruginosa* was

only analyzed by eye comparison as amplification was achieved only in two annealing temperature.
